# Combination of Dichloroacetate and Atorvastatin Regulates Excessive Proliferation and Oxidative Stress in Pulmonary Arterial Hypertension Development via p38 Signaling

**DOI:** 10.1155/2020/6973636

**Published:** 2020-06-11

**Authors:** Tangzhiming Li, Suqi Li, Yilu Feng, Xiaofang Zeng, Shaohong Dong, Jianghua Li, Lihuang Zha, Hui Luo, Lin Zhao, Bin Liu, Ziwei Ou, Wenchao Lin, Mengqiu Zhang, Sheng Li, Qiuqiong Jiang, Qiangqiang Qi, Qingyao Xu, Zaixin Yu

**Affiliations:** ^1^Department of Cardiology, Shenzhen People's Hospital, The First Affiliated Hospital of Southern University of Science and Technology, The Second Clinical Medical College of Jinan University, Guangdong, China; ^2^Department of Cardiology, Xiangya Hospital, Central South University, Changsha, China; ^3^State Key Laboratory of Cardiovascular Disease, Fu Wai Hospital, National Center for Cardiovascular Diseases, Chinese Academy of Medical Sciences and Peking Union Medical College, Beijing, China; ^4^National Clinical Research Center for Geriatric Disorders, Xiangya Hospital, Central South University, 87 Xiangya Road, Changsha, Hunan, China; ^5^Centre for Pharmacology and Therapeutics, Division of Experimental Medicine, Imperial College London, Hammersmith Hospital, London W12 0NN, UK

## Abstract

Pulmonary arterial hypertension (PAH) is a lethal disease generally characterized by pulmonary artery remodeling. Mitochondrial metabolic disorders have been implicated as a critical regulator of excessively proliferative- and apoptosis-resistant phenotypes in pulmonary artery smooth muscle cells (PASMCs). Dichloroacetate (DCA) is an emerging drug that targets aerobic glycolysis in tumor cells. Atorvastatin (ATO) is widely used for hyperlipemia in various cardiovascular diseases. Considering that DCA and ATO regulate glucose and lipid metabolism, respectively, we hypothesized that the combination of DCA and ATO could be a potential treatment for PAH. A notable decrease in the right ventricular systolic pressure accompanied by reduced right heart hypertrophy was observed in the DCA/ATO combination treatment group compared with the monocrotaline treatment group. The DCA/ATO combination treatment alleviated vascular remodeling, thereby suppressing excessive PASMC proliferation and macrophage infiltration. In vitro, both DCA and ATO alone reduced PASMC viability by upregulating oxidative stress and lowering mitochondrial membrane potential. Surprisingly, when combined, DCA/ATO was able to decrease the levels of reactive oxygen species and cell apoptosis without compromising PASMC proliferation. Furthermore, suppression of the p38 pathway through the specific inhibitor SB203580 attenuated cell death and oxidative stress at a level consistent with that of DCA/ATO combination treatment. These observations suggested a complementary effect of DCA and ATO on rescuing PASMCs from a PAH phenotype through p38 activation via the regulation of mitochondrial-related cell death and oxidative stress. DCA in combination with ATO may represent a novel therapeutic strategy for PAH treatment.

## 1. Introduction

Pulmonary arterial hypertension (PAH) is characterized by the remodeling of precapillary pulmonary arteries, leading to the increase in pulmonary vascular resistance and eventually right heart failure if left untreated [1]. Excessive proliferation of pulmonary artery smooth muscle cells (PASMCs) has been identified as the hallmark of promoting this pulmonary vascular change [2]. However, no specific therapeutic approach to date has been successfully translated into clinical practice in PAH. Vasodilators remains the mainstay of currently licensed PAH treatments [3, 4], although they have been proved inadequate in reversing PASMC proliferation while also being prohibitively expensive [5]. Therefore, discovering novel pathobiology mechanisms is crucial in developing new drugs or repurposing existing drugs for successful PAH treatment.

Mitochondrion dysfunction has been theorized as a crucial player in PAH development by increasing the production of reactive oxygen species (ROS) through the activity of the mitochondrial electron transport chain [6]. The metabolic shift of mitochondria, known as “Warburg effect,” is related to the hyperpolarization of the mitochondrial membrane [7] and accompanied by resistance to apoptosis [8]. Compelling evidence supports the hypothesis that correction of metabolic abnormalities could suppress PAH development [9–11].

Dichloroacetate (DCA) is an analog of pyruvate that inhibits mitochondrial pyruvate dehydrogenase kinase [12] and enhances oxidative phosphorylation [13]. DCA is used to treat several types of solid tumors [14–16] and PAH [7, 9–11, 17–19] owing to its ability to restore aerobic glycolysis. DCA is speculated to prevent or reverse established monocrotaline- (MCT-) induced PAH in rats [17]. However, DCA only has a moderate inhibition effect on PAH development, implying that it may not be sufficient to suppress PAH pathologies. Statins are a class of drugs that inhibit 3-hydroxy-3-methylglutaryl Co-A reductase. Several experiments have demonstrated that statins attenuate the development of PAH models [20, 21]. The effect of reducing hypercholesterolemia may help in metabolic regulation; moreover, statin therapy for PAH has been evaluated in randomized controlled trials for simvastatin [22, 23] and atorvastatin (ATO) [5]. However, none of these clinical studies provided evidence of an improvement in patients' long-term prognoses.

DCA has been shown to reverse resistance to the antiapoptotic and hyperproliferative susceptibility of PASMCs by suppressing the Warburg effect, resulting in the reversal of PAH vascular remodeling. ATO has been approved for the treatment of cardiovascular diseases that have the potential for rapid translation to PAH. Given that DCA and ATO have potent inhibition effects on PAH development by metabolic regulation, we hypothesized that combined therapy with DCA and ATO would reverse MCT-induced pulmonary vascular remodeling by attenuating the hyperproliferative and antiapoptotic phenotypes of PASMCs.

## 2. Materials and Methods

### 2.1. Animal Experiments

All experiments were approved by the Institutional Animal Care and Use Committee of Central South University. Adult male Sprague–Dawley (SD) rats (8–10 weeks old) were matched according to weight (200–250 g). The rats were randomly assigned to one of the five groups (*n* = 5 for each group): (1) control group, saline injection with normal drinking and food; (2) MCT group, MCT injection with normal drinking and food; (3) MCT+DCA group, MCT injection with DCA treatment (drinking water, 70 mg/kg per day, Sigma-Aldrich, USA); (4) MCT+ATO group, MCT injection with ATO treatment (oral gavage, 10 mg/kg per day, Pfizer, USA); and (5) MCT+DCA+ATO group, MCT injection with DCA and ATO treatment. Saline was given to the control group as placebo. For the other four groups, MCT (60 mg/kg) [24, 25] was injected into the subcutaneous tissue to induce progressive pulmonary arterial hypertension at the end of 21 days.

### 2.2. Hemodynamic Measurements and Tissue Collection

At the end of the experiment, the rats were deeply anesthetized with a lethal dose of sodium pentobarbital (50 mg/kg i.p). Right ventricular systolic pressure (RVSP) was measured through the right jugular vein by a precurved catheter. Hemodynamic data were recorded and analyzed with a PowerLab Data Acquisition system (AD Instruments) [24]. The right lung and heart tissues were flushed with saline to clear blood and snap frozen in liquid nitrogen and stored at –80°C in preparation for Western blot analysis. Hearts were dissected and weighed; the ratio of the right ventricle to the left ventricle plus the septum (RV/[LV+S]) was used as an index of RV hypertrophy. The left lung was fixed in 4% paraformaldehyde solution and embedded in paraffin for histological examination.

### 2.3. Morphometry Analysis, Immunohistochemistry, and Immunofluorescence

Formaldehyde-fixed and paraffin-embedded lung tissue sections were stained with hematoxylin and eosin (H&E staining). Morphometric analyses were performed on pulmonary arteries with an external diameter between 50 and 100 *μ*m. Medial thickness was calculated using the following formula: medial thickness (%) = medial wall thickness/external diameter × 100 [24, 26]. For quantitative analyses, 30 small pulmonary vessels from each animal that were less than 50 *μ*m in external diameter were evaluated for muscularization [24]. Elastic staining was conducted according to the manufacturer's protocol (Sigma-Aldrich® Elastic Stain Procedure No. HT25). For immunohistochemistry examination, lung sections were stained for anti-Ki67 and anti-CD68 to evaluate proliferation and inflammation in each group.

### 2.4. PASMC Isolation and Culture

Segments of pulmonary artery of the SD rats were cut to expose the luminal surface. The endothelium was removed by gentle scraping, and the media were peeled away from the underlying adventitial layer. After isolation, a modified collagenase digestion protocol was employed. PASMCs were incubated in a medium containing 0.5 mg/ml collagenase type I (Worthington Biochemical Corporation). The explants were incubated in DMEM supplemented with 20% FBS until the cells formed confluent monolayers [2, 27]. After reaching confluency, the cells were passaged in 0.25% trypsin, maintained in culture in DMEM supplemented with 10% FBS, and maintained at 37°C in 5% CO_2_. The cells up to passage five were used for experiments. The cells were stained for *α*-smooth muscle actin by immunocytochemistry. PA smooth muscle cells were placed in a serum-free medium for 24 h and then exposed to dichloroacetate (DCA, 5 mM, dissolved in PBS) (Sigma-Aldrich, Lyon, France) and ATO (1 or 10 mM, dissolved in DMSO) (R&D Systems, Lille, France) for 24 h.

### 2.5. Cell Viability Assay

Cell viability was detected using a CCK-8 Kit (Dojindo, Japan) according to the manufacturer's instructions. The absorbance was measured at 450 nm.

### 2.6. Mitochondrial Membrane Potential, Mitochondrial Reactive Oxygen Species, and Intracellular Reactive Oxygen Species Detection

Mitochondrial membrane potential (MMP) was assessed using the fluorescent probe JC-1 (C2006, Beyotime Biotech, China). PASMCs of each group were incubated with 5 *μ*mol/l MitoSOX Red (407778ES50, Yeasen Biotech, China) in HBSS to measure mitochondrial ROS. Superoxide (O_2_^·–^) production was measured with dihydroethidium (DHE, S0063, Beyotime Biotech, China) as previously described [28]. The cultured PASMCs of each group were enriched by O_2_^·–^ and stained by DHE (2 mM).

The samples were left in the fluorescent probe for 30 min at 37°C and then washed to remove unbound dye. The samples were immediately monitored under a fluorescence microscope (Nikon, Tokyo, Japan) in a dark room. Mean JC-1, MitoSOX, and DHE fluorescence intensity was captured by dividing individual object fluorescence intensities and is expressed in arbitrary fluorescence units (A. U.). The experiments were performed in triplicate.

### 2.7. Western Blotting and Quantification

After the indicated duration of treatment, lung tissue samples and cells were homogenized in lysis buffer containing Roche complete protease inhibitor cocktail (Roche, Basel, Switzerland). Concentration-normalized protein samples were prepared with SDS loading buffer. About 20–30 *μ*g of total protein was separated on 12% polyacrylamide gels and transferred onto polyvinylidene fluoride membranes [29]. The membranes were then blocked and probed with one of the following primary antibodies: anti-GRP78 (1 : 1,000, #ab108613, Abcam, USA), anti-CHOP (1 : 1,000, #ab11419, Abcam, USA), anti-Bax (1 : 1,000, 50599-2-Ig, Proteintech, USA), and anti-Bcl2 (1 : 1,000, 12789-1-AP, Proteintech, USA). As a loading control, all blots were reprobed with an antibody toward anti-beta actin (1 : 1,000, #ab8827, Abcam, USA). Densitometry analysis was performed using ImageJ software.

### 2.8. Flow Cytometry Analysis of Cell Cycle

Cell cycle distribution was determined by staining DNA with propidium iodide (PI). The cells were collected, washed with ice-cold PBS (pH 7.4) buffer twice, fixed with 70% alcohol at 4°C overnight, and then stained with PI (20x) in the presence of RNase A (50x) for 30 min at least. The percentages of cells in different cell cycle phases were measured using a flow cytometer (Beckman Coulter Epics), and the percentages of cells in the G0/G1, S, and G2/M phases were analyzed with the ModiFit LT 5.0 software.

### 2.9. Flow Cytometry Analysis of Apoptosis

Flow cytometry analysis of PASMC apoptosis was conducted using annexin V/PI staining. The extent of apoptosis was measured with the annexin V-FITC apoptosis detection kit (Beyotime, China) as described by the manufacturer's instruction. The samples were then analyzed with FlowJo software.

### 2.10. Statistical Analysis

Student's *t*-tests were used for comparisons between two groups. Multiple comparisons were assessed by one-way ANOVA, followed by the appropriate post hoc test for significance. All statistical tests used two-sided tests of significance. All data are reported as mean ± SD. *P* < 0.05 was considered statistically significant. Data analysis was performed using SPSS 20 (IBM SPSS Inc., Chicago, USA), and figures were prepared using GraphPad Prism 6.0 software.

## 3. Results

### 3.1. Combination of DCA and ATO Attenuated PAH Hemodynamic Disorder and Right Heart Hypertrophy

**Figure 1:**
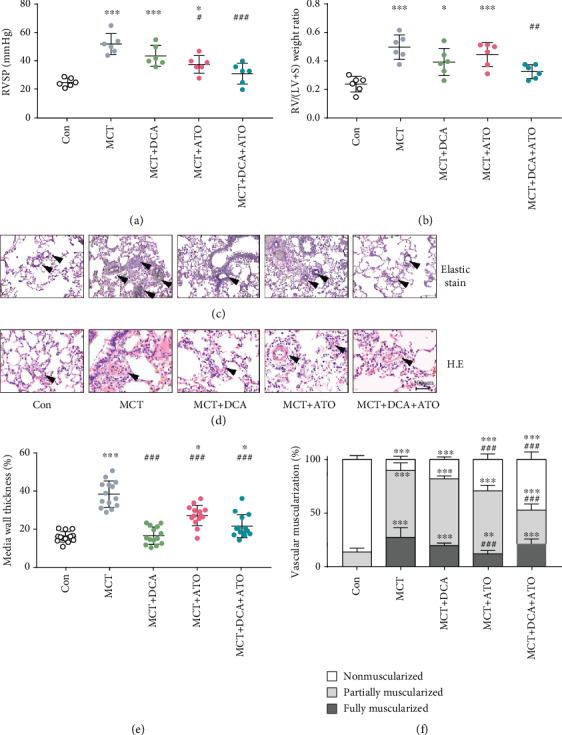
Pulmonary arterial hypertension induced by MCT is inhibited by dichloroacetate and atorvastatin combination therapy. (a, b) Measurement of RVSP and right ventricular hypertrophy in each treatment group (*n* = 6). (c, d) Representative images of pulmonary artery remodeling in each group; arrows indicate representative vessels. (c) Elastic Van Gieson staining and (d) Hematoxylin and eosin staining. Scale bars, 100 *μ*m. (e, f) Quantification of remodeled vessels. (e) Medial wall thickness (*n* = 15) and (f) percentage of nonmuscularized or partially muscularized or fully muscularized arteries at alveolar and duct levels (*n* = 30). ^∗^ and ^∗∗∗^ indicate *P* < 0.05 and *P* < 0.001, respectively, comparing the control group; ^#^, ^##^, and ^###^ indicate *P* < 0.05, *P* < 0.01, and *P* < 0.001, respectively, comparing the MCT group. MCT: monocrotaline; RVSP: right ventricular systolic pressure; RV/(LV+S): right ventricular/(left ventricular + septum); H&E: hematoxylin and eosin staining. Bars represent mean ± SEM.

DCA or ATO was given separately and in combination to MCT-treated rats to assess their effects in a PAH model. The DCA and ATO combination treatment significantly restored the right ventricular systolic pressure and right heart remodeling in MCT rats while monotherapy of DCA or ATO only presented a marginal reversal of hemodynamic change or right ventricular hypertrophy (Figures 1(a) and 1(b)).

We then determined vascular remodeling by Elastic Van Gieson staining and H.E. staining. Thickened intima and medium layers of pulmonary vessels were noted in MCT-induced PAH rats, which was moderately modified by separate treatments of DCA or ATO but significantly ameliorated in the combination group (Figures 1(c)–1(e)). Quantification of vascular muscularization revealed MCT increased the ratio of partially and fully muscularized vessels and reduction of peripheral arterial volume was restored by DCA or ATO at different levels. By contrast, the DCA/ATO combination treatment induced a remarkable increase in normal vascular ratio and a decrease in partially occluded vessels (Figure 1(g)).

Collectively, these results demonstrated that DCA and ATO play different roles in regulating disordered hemodynamics, and combining both of them can lead to histological improvement in PAH.

### 3.2. Combination of DCA and ATO Was Superior in Proliferation and Inflammatory Suppression

**Figure 2:**
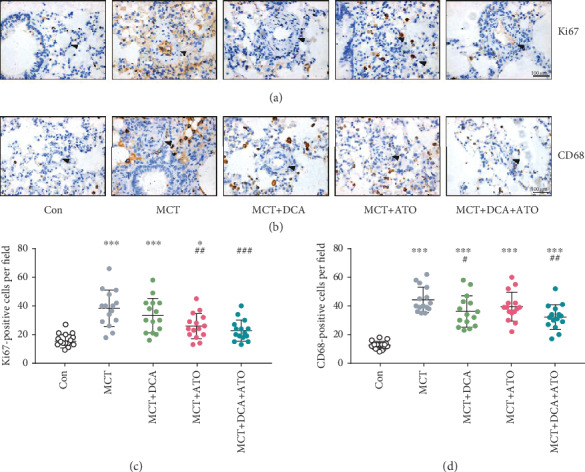
Cell proliferation and macrophage infiltration in PAH were reduced by dichloroacetate and atorvastatin combination treatment. (a, b) Representative immunohistochemistry images of proliferation and inflammation status of each group. Arrows indicate morphological structures of pulmonary arteries after indicated treatment. Scale bars, 100 *μ*m. (c, d) Semiquantitative (c) Ki67- and (d) CD68-positive cells in high-resolution fields of view (*n* = 15). ^∗^ and ^∗∗∗^ indicate *P* < 0.05 and *P* < 0.001, respectively, comparing the control group; ^#^, ^##^, and ^###^ indicate *P* < 0.05, *P* < 0.01, and *P* < 0.001, respectively, comparing the MCT group.

Pulmonary vascular proliferation and inflammation infiltration in each group of rats were investigated. The DCA/ATO combination treatment elicited a better effect in suppressing the Ki67 expression (Figures 2(a) and 2(c)) and CD68 infiltration (Figures 2(b) and 2(d)) in peripheral vessels than the control group. This result was consistent with that described in Figure 1. DCA and ATO exhibited a complementary effect on PAH pathology suppression.

### 3.3. DCA Reduced PASMC Viability and Enhanced Viability when Combined with ATO without Stimulating Further Cell Damage

Primary cultures were isolated from the pulmonary arteries to determine the inhibitory effects of DCA and ATO on PASMC proliferation (Supplementary Figure 1). PASMCs were exposed to 30 *μ*g/ml PDGF-BB for further experiments (Supplementary Figure 2).

**Figure 3:**
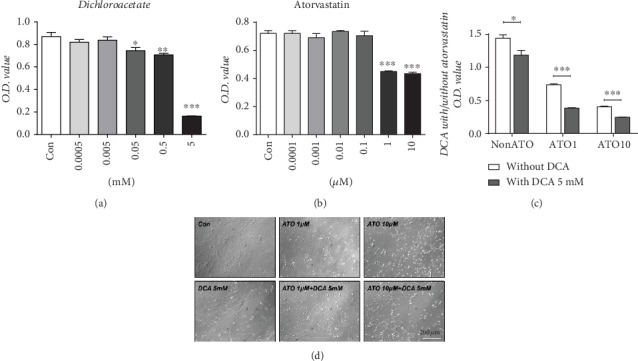
PASMC proliferation in response to prohypertensive growth factor was inhibited by dichloroacetate and atorvastatin exposure. (a, b) Cell viability assay in PASMCs exposed to (a) dichloroacetate (*n* = 7) and (b) atorvastatin (*n* = 7). ^∗^, ^∗∗^, and ^∗∗∗^ indicate *P* < 0.05, *P* < 0.01, and *P* < 0.001, respectively, comparing the control group. (c) Cell viability assay in PASMCs treated with different concentrations of atorvastatin with or without 5 mM dichloroacetate (*n* = 5). ^∗^ and ^∗∗∗^ indicate *P* < 0.05 and *P* < 0.001, respectively, comparing the non-DCA group. (f) White field of PASMCs showed characteristic morphological changes. Scale bar: 100 *μ*m.

When PASMCs were treated with DCA and ATO separately, cell viability decreased in a concentration-dependent manner (Figures 3(a) and 3(b)). Therefore, CCK-8 assay was performed on PDGF-stimulated PASMCs treated with DCA with or without ATO. Compared with DCA monotherapy, the combination treatment induced a marked decrease in cell proliferation (Figure 3(c)). According to the morphological appearance of the PASMCs (Figure 3(d)), the single DCA treatment motivated a moderate proapoptotic effect. Conversely, ATO-exposed cells typically became rounded and isolated from their neighbors. These changes were restored by DCA cotreatment, which preserved the morphological structures of the cells, i.e., dual combination may result in cell death through a more controlled manner (Figure 3 (d)).

### 3.4. DCA and ATO Combination Treatment Modified Mitochondrion-Related Oxidative Stress in Proliferative PASMCs

**Figure 4:**
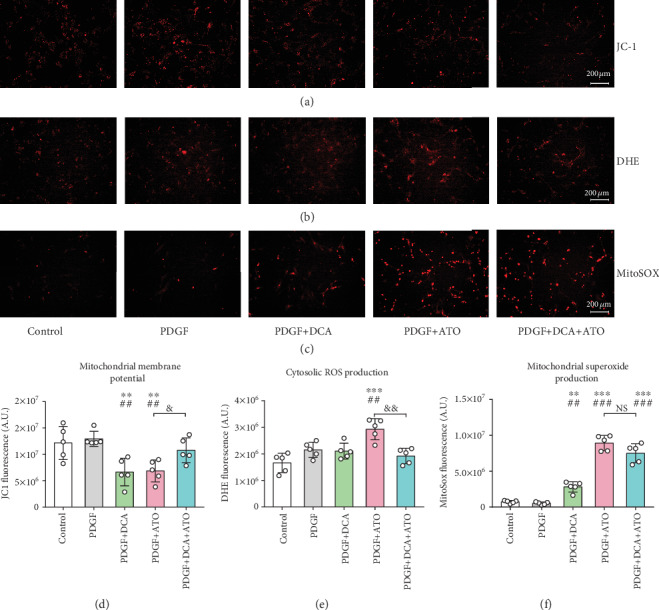
Dichloroacetate and atorvastatin regulated mitochondrial alterations in PASMCs. (a) Mitochondrial membrane potential (MMP) evaluated using the JC-1 probe. (b) Cytosolic ROS production detected using the DHE fluorescence probe. (c) Mitochondrial superoxide expressed by MitoSOX fluorescence probe. (d–f) Semiquantitation of MMP (d), cytosolic reactive oxygen species (e), and mitochondrial superoxide (f) (*n* = 5). ^∗∗^ and ^∗∗∗^ indicate *P* < 0.01 and *P* < 0.001, respectively, comparing the control group; ^##^ and ^###^ indicate *P* < 0.01 and *P* < 0.001, respectively, comparing the PDGF group. ^&^ and ^&&^ indicate *P* < 0.05 and *P* < 0.01, PDGF+ATO compared with PDGF+DCA+ATO. Scale bars: 200 *μ*m.

To determine the underlying cytotoxicity mechanisms of DCA and ATO, we analyzed mitochondrion-associated cell death by using the redox-sensitive probe JC-1. Separate DCA and ATO treatments decreased MMP, implying that these two drugs are capable of leading to proliferative SMC apoptosis. However, when DCA and ATO were combined, MMP increased compared with either treatment alone (Figures 4(a) and 4(d)).

Cytosolic ROS and mitochondrial superoxide production were measured by DHE and MitoSOX, respectively (Figures 4(b) and 4(c)). Consistent with JC-1 detection, DCA only slightly upregulated mitochondrial superoxide production compared with ATO, indicating that severe mitochondrion-associated cell damage was caused by ATO exposure. The substantial increases in cytosolic ROS caused by ATO were prevented in the DCA/ATO combination treatment group. A similar trend was observed in mitochondrial ROS detection, where DCA attenuated ATO-induced mitochondrial superoxide production (although the effect was not statistically significant) (Figures 4(e) and 4(f)).

### 3.5. DCA and ATO Combination Treatment Balanced Apoptosis Proliferation and Endoplasmic Reticulum Stress Disorder in PASMCs

**Figure 5:**
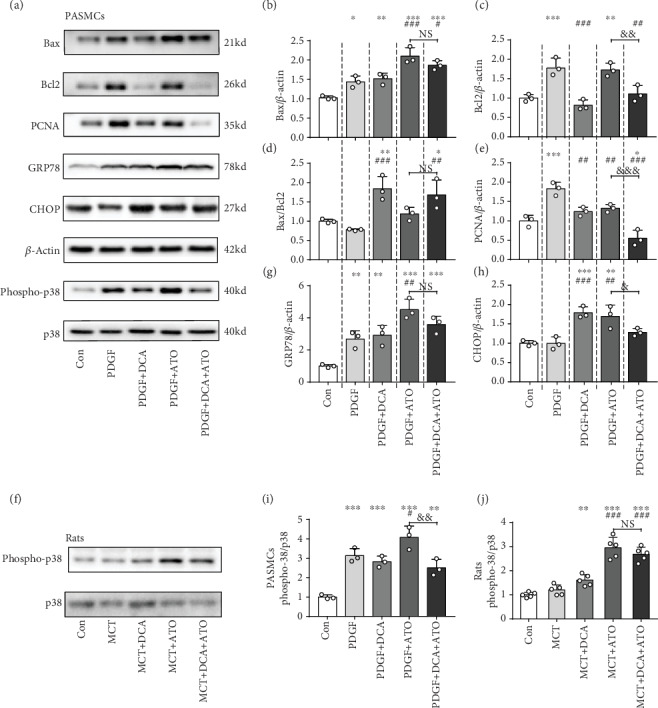
Dichloroacetate and atorvastatin regulated the balance of proliferation and apoptosis and endoplasmic reticulum stress accompanied by p38 activation. Representative immunoblots and relative densitometric analysis of proliferation, apoptosis marker, and endoplasmic reticulum stress-associated marker. (a) PASMCs exposed to PDGF, DCA, or ATO as indicated above compared with the controls (*n* = 3). (b–e, g–i) Immunoblot quantifications of Bax, Bcl2, Bax/Bcl2, PCNA, GRP78, CHOP, and phospho-p38/total p38 of each group (*n* = 3). (f, j) Immunoblot phospho-p38 and total p38 quantification in the PASMCs (*n* = 3) and rat model (*n* = 5). *β*-Actin was used as a loading control. ^∗^, ^∗∗^, and ^∗∗∗^ indicate *P* < 0.05, *P* < 0.01, and *P* < 0.001, respectively, compared with the control group; ^#^, ^##^, and ^###^ denote *P* < 0.05, *P* < 0.01, and *P* < 0.001, respectively, comparing the PDGF group. ^&^, ^&&^, and ^&&&^ represent *P* < 0.05, *P* < 0.01, and *P* < 0.001, the PDGF+ATO group compared with the PDGF+DCA+ATO group.

We further determined the role of DCA and ATO in proliferation and apoptosis balance. Established proliferative PASMCs were harvested to measure Bax, Bcl2, and PCNA expression. DCA increased apoptosis, whereas ATO upregulated Bax and Bcl2 expression. However, the net effect on Bax/Bcl2 increase was not different from that of the control group (Figures 5(a)–5(d)). DCA did not attenuate cytotoxicity when the combination treatment was applied (Figure 5(d). In addition, both DCA and ATO inhibited cell proliferation, and the combination treatment led to a considerable reduction in PCNA expression (Figures 5(a) and 5(e)). Additionally, we compared the effects of ATO monotherapy and DCA/ATO combination treatment on Bax, Bcl2, and PCNA expression. We found that the combination treatment further attenuated antiapoptosis and proliferation compared with ATO monotherapy.

We analyzed the effects of DCA and ATO on oxidative stress by using a fluorescence probe and cytosolic stress (endoplasmic reticulum stress, ERS) biomarker. DCA and ATO exhibited the capacity to activate the ERS of PASMCs, especially ATO. DCA prevented ATO-induced ERS-related cellular damage (Figures 5(a), 5(g), and 5(h)), implying that DCA relieved ATO's prooxidative effect. Similar results were observed in p38 signaling. DCA ameliorated ATO-induced p38 activation in PDGF-BB-pretreated PASMCs. DCA also partly (but not significantly) restored ATO-related p38 upregulation in MCT rats.

Collectively, these results provided evidence that DCA prevented ATO-induced overoxidative stress in which p38 may participate. DCA plus ATO effectively reduced PASMC proliferation and enhanced apoptosis.

### 3.6. p38 Activation Was Involved in DCA- and ATO-Related Apoptosis and Proliferation in PASMCs

**Figure 6:**
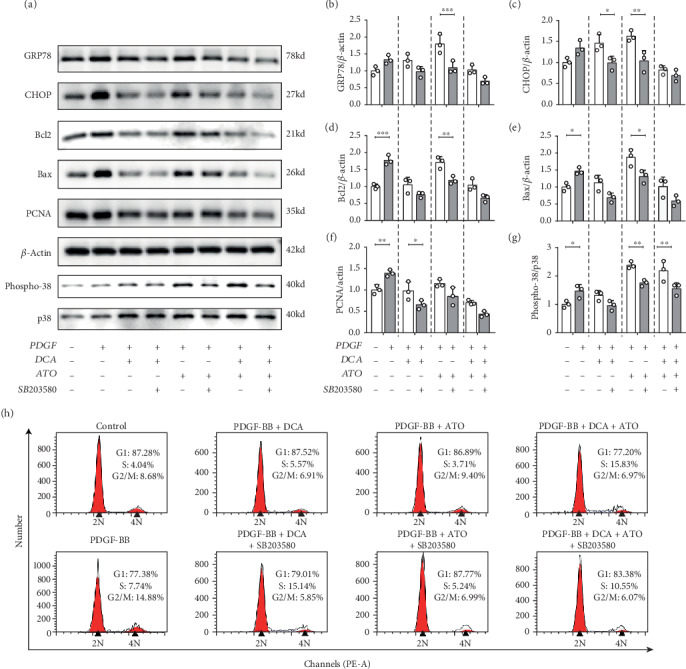
p38 inhibition alleviated dichloroacetate- and atorvastatin-related endoplasmic reticulum stress and apoptosis/antiapoptosis and proliferation disorders. Representative immunoblots and relative densitometric analysis of proliferation, apoptosis marker, and endoplasmic reticulum stress-associated marker. (a) PASMCs exposed to PDGF, DCA, and ATO with or without p38 inhibitor as indicated above (*n* = 3). (b–g) Immunoblot quantifications of related markers (*n* = 3). (H) Flow cytometry analysis of indicated exposure to PASMCs on cell cycle distribution. *P* < 0.05, *P* < 0.01, and *P* < 0.001. SB203580: p38-specific inhibitor.

To gain additional perspective on p38 in DCA and ATO treatments, we sought to target this pathway by using the p38-specific inhibitor. DCA and ATO monotherapy and DCA/ATO combination therapy of PASMCs were treated with SB203580. These therapies inhibited p38 phosphorylation (Figures 6(a) and 6(g)), resulting in the reduction of endoplasmic reticulum stress (Figures 6(a)–6(c)). As expected, SB203580 downregulated Bcl2 and Bax expression, suggesting that ATO-mediated antiapoptosis and apoptosis balance was p38 dependent, at least partially. Simultaneously, PCNA expression was found to be also related to p38 inhibition, and SB203580 reduced the effects of DCA on cell growth inhibition.

**Figure 7:**
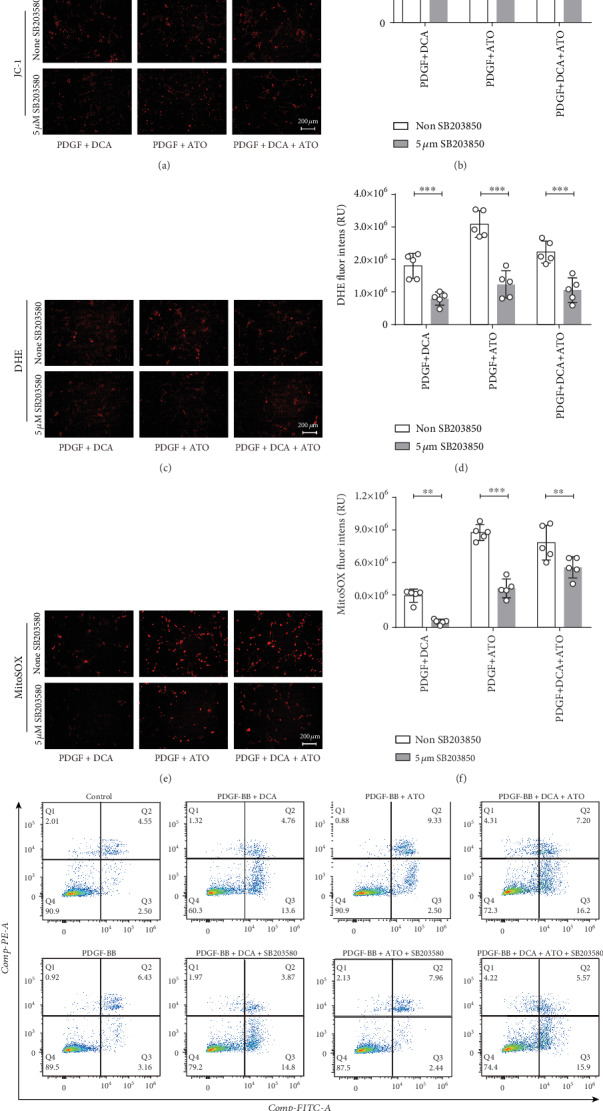
p38 inhibitor reduced dichloroacetate- and atorvastatin-related cytosolic and mitochondrial reactive oxidative stress accompanied by apoptosis suppression. (a, b) Mitochondrial membrane potential evaluated using a JC-1 probe and its semiquantitation. (c, d) Cytosolic ROS production detected using the DHE fluorescence probe and its measurement. (e, f) Mitochondrial superoxide expressed by MitoSOX fluorescence probe and its assessment. Scale bars: 200 *μ*m (*n* = 5). ^∗∗^ and ^∗∗∗^ indicate *P* < 0.01 and *P* < 0.001, respectively. Analyses performed by unpaired Student's *t*-test. Bars represent mean ± SEM. (g) Flow cytometry analysis of PASMC apoptosis induced by indicated treatment using annexin V/PI staining. SB203580: p38-specific inhibitor.

We investigated the effects of DCA and ATO with or without SB203580 on cell cycle regulation (Figure 6(h)). Cell cycle distribution in the PASMCs was considerably modified by PDGF pretreatment, advancing the cells from the G1 and S phases to G2/M phase. DCA and ATO abolished the effects of PDGF on proliferation enhancement. DCA and ATO treatment in conjunction with SB203580 resulted in a greater percentage of the cell population in the S phase compared with that in single drug delivery, suggesting that p38 inhibition led to greater cell proliferation and reversed DCA/ATO cell growth inhibition.Cell growth and apoptosis are accompanied by ROS and ERS conditions in proliferative PASMCs, as we demonstrated above. Herein, we exposed PDGF-pretreated PASMCs to DCA and ATO with or without SB203580 for MMP and oxidative stress evaluation. P38 inhibition did not affect MMP when DCA and ATO were given separately, but the combination treatment restored MMP (Figures 7(a) and 7(b)). Moreover, SB203580 substantially reduced cytosolic ROS and mitochondrial superoxide production caused by DCA and ATO (Figures 7(c)–7(f)). Hence, regulation of growth and apoptosis was closely related to p38 activation and its resulting oxidative stress.

Lastly, annexin V-FITC and a PI staining flow cytometry apoptosis detection kit were utilized to examine the influence of the two drugs on PASMC cytotoxicity. As shown in Figure 7, apoptotic PASMCs greatly increased in the DCA, ATO, and DCA+ATO groups. When SB203580 was added to the drug treatment groups, only ATO and DCA+ATO showed a reduction in apoptotic cell population, although SB203580 mediated a moderate apoptosis decrease. These results also supported our previous findings discussed above.

## 4. Discussion

**Figure 8:**
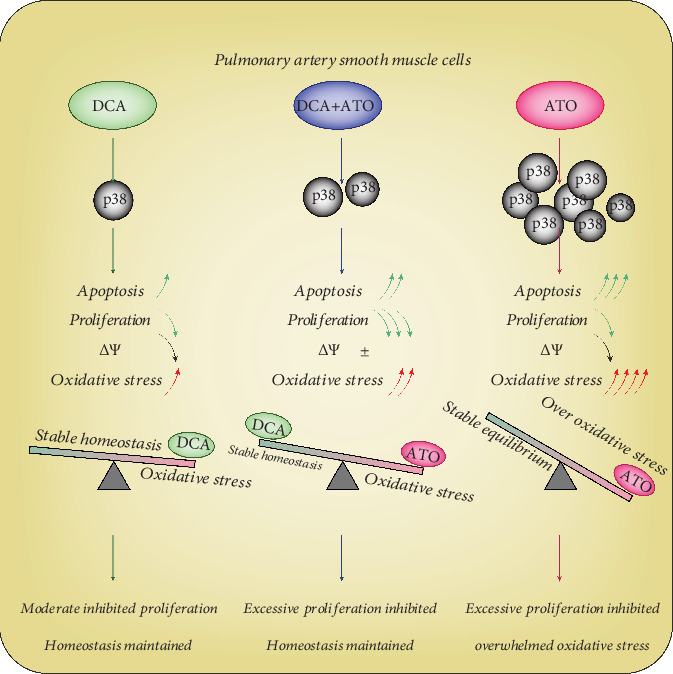
A schematic showing the proposed mechanism of dichloroacetate- and atorvastatin-related phenotype regulation. DCA inconsiderably increased apoptosis and oxidative stress and decreased proliferation and *ΔΨ*_m_ (MMP) in a p38 activation-dependent manner. By contrast, ATO markedly upregulated the p38 signal pathway and oxidative stress, resulting in profound apoptosis. Slightly reduced proliferation and MMP when DCA and ATO combination treatment could achieve more therapeutic targets and only cause moderate ROS damage.

In the present study, we demonstrated that the DCA/ATO combination therapy was more efficient in inhibiting PASMC proliferation, correcting apoptosis resistance, and reducing an overwhelmed oxidative stress phenotype than monotherapy. This concept (Figure 8) was based on the findings that (1) DCA and ATO positively altered the phenotype of the PAH rat model, (2) DCA and ATO worked collaboratively in inhibiting PASMC viability, (3) DCA- and ATO-induced cell death was accompanied by p38-dependent ROS production, and (4) DCA alone was capable of suppressing cell proliferation; however, when ROS and cell apoptosis was great, DCA prevented excessive oxidative stress and cell injury.

These experiments are our first attempt to combine glucose- and cholesterol-modulating drugs in treating PAH. DCA and ATO combination treatment prevented vascular cell hyperproliferation and macrophage infiltration (Figure 2), which are the hallmarks of PAH pathogenesis. Consequently, we demonstrated that DCA or ATO monotherapy exhibited a dose-dependent cytotoxic manner, whereas the combination treatment conferred greater and more substantial suppression of cell viability than monotherapy, indicating that these drugs have a reciprocal effect on proliferation inhibition. In a PAH rat model, ATO administration is associated with protective effects, including a reduction in pulmonary thickness [30–32] and right heart hypertrophy [33]. We also found that ATO administration resulted in the apoptosis of PASMCs, as their morphology became round and isolated from their neighbors. Intriguingly, DCA in combination with ATO resulted in a remarkable decrease in viability while relatively preserving cell morphology, implying that coadministration of both drugs could prevent cell proliferation in a comparably controlled manner (Figure 3(d)). This observation was consistent with that of a recent study that showed that DCA and cisplatin combination treatment retained their anticancer properties while inducing better effects on preventing cisplatin-induced nephrotoxicity [34].

Thus, we clarified the underlying mechanism by which DCA and ATO reduce proliferation and revealed their reciprocal effects on reducing cellular toxicity. ATO led to greater cytosolic ROS and mitochondrial superoxide production than DCA, whereas DHE and MitoSOX fluorescence density was relatively restored in the combination treatment group. DCA was previously reported to contribute to cell protection by attenuating oxidative stress [34]. In the present study, DCA either alone or in combination with ATO presented a bidirectional role in maintaining the balance of cell proliferation and apoptosis, making it a potential approach in PAH treatment.

p38 activation has a potent role in PAH pathobiology [31, 35, 36]. p38 signaling plays a crucial role in the production of proinflammatory cytokines [37]. p38 inhibition by SB203580 can prevent PAH development by reversing RVSP and right heart hypertrophy [36]. p38 is a well-known redox-sensitive kinase involved in vascular diseases [35]. As a regulator of apoptosis, p38 is activated upon phosphorylation often in response to cell stress and ROS. Multiple studies have implicated oxidative stress in PAH development [38, 39]. ROS modulates vascular force and tone, thereby regulating cellular proliferation and apoptosis and PAH pathological processes. Suppression of mitochondrial activities, such as glucose oxidation, which results in metabolic switch to glycolysis, contributes to an antiapoptotic phenotype [40]. Therefore, maintaining oxidative stress at a proper level is essential for PAH treatment. The mitochondria are the key source of vascular oxidative stress in vessel dysfunction [41]. Therefore, we investigated p38-dependent cell death and ROS production following DCA and ATO treatment. In the present study, we demonstrated that DCA and ATO exerted their PASMC inhibition effect in a p38-dependent manner (Figures 5 and 6). In DCA- and ATO-treated PASMCs, p38 activation was associated with different treatment strategies (Figures 5(a) and 5(i)). Coadministration of these drugs resulted in a notable reduction in p38 phosphorylation compared with the ATO-treated group. A similar trend was observed *in vivo*. However, p38 phosphorylation moderately decreased in the DCA/ATO combination treatment group compared with ATO-treated group (Figures 5(f) and 5(j)). Nevertheless, we recognize that numerous factors were at play in the animal experiments. We believe that amplification of the rat samples would be helpful in achieving a statistical difference.

DCA has the ability to reverse pulmonary artery remodeling and improve right heart function and survival by reversing aerobic glycolysis [7, 42]. Mitochondrial abnormalities are attracting increased attention in the PAH treatment. In the present study, we demonstrated that DCA could reduce MMP and alleviate oxidative stress induced by ATO. This result was consistent with that of our previous studies. Statins can reportedly induce apoptosis of neointimal smooth muscle cells [43], reduce MCT-induced PAH [44] and chronic hypoxia PAH [45], and prevent pulmonary artery muscularization [21]. We found that ATO had a better effect in preventing RVSP elevation and Ki67 upregulation than DCA. However, we believe that PASMC apoptosis caused by statin administration, through oxidative stress buildup both *in vivo* and *in vitro*, could abolish its protective effects.

In the present study, we reviewed previous studies and presented a potential strategy for the combination therapy of PAH. Markedly reduced right ventricular systolic pressure and right heart hypertrophy were observed in the DCA and ATO combination treatment group, and this result may correspond to reduced vascular remodeling. DCA and ATO exhibited complementary effects on rescuing from a PAH phenotype.

However, several limitations of the current study should be mentioned. First, although the p38-specific inhibitor SB203580 would reportedly not affect oxidative stress or proliferation by itself [46, 47], whether SB203580 could induce ROS without DCA or ATO delivery should be investigated. In addition, we did not conduct p38 genetic depletion in the animal experiments. A previous study reported that p38 inhibition alleviates PAH development [36]. The present results were consistent with this conclusion. Finally, further studies should evaluate the safety of DCA/ATO combination treatment. Several previous works stated that DCA and ATO are relatively safe for patients. ATO dosages ranging from 10 mg to 40 mg daily rarely result in adverse events [5]. DCA dosages of 3–12.5 mg/kg are approved for PAH treatment (ClinicalTrials.gov NCT01083524) [11], with no clinically significant change in QT intervals of electrocardiogram, cardiac rhythm, and liver, bone marrow, and renal functions.

Accumulating evidence indicates that appropriate combinations of multiple classes of drugs that target different pathogenic pathways may improve clinical outcomes [48–51]. The use of applicable combination therapies is not well documented, but it is a promising prospect for broadening PAH treatment strategies. We based our hypotheses on observations that metabolic disorders are a crucial underlying mechanism in PAH development. We then focused on the properties of DCA and ATO in regulating glucose and cholesterol separately. We found that the combination of these drugs had a superior effect in inhibiting PAH phenotypes. The novel combination is a clinically relevant finding that may provide new insights for PAH treatment.

## 5. Conclusions

The combination of DCA and ATO reduced excessive proliferation and promoted apoptosis and reduced oxidative stress in a controlled manner that is dependent on p38 activation.

## Data Availability

The data that support the findings of this study are openly available.

## Supplementary Materials

Supplementary Materials Supplementary Figure 1: PASMC isolation and identification. Supplementary Figure 2: PASMCs were starved for 24 h following indicated stimulation methods.
