# Supplementary figures and images for: Combination of Dichloroacetate and Atorvastatin Regulates Excessive Proliferation and Oxidative Stress in Pulmonary Arterial Hypertension Development via p38 Signaling
Source: Oxid Med Cell Longev. 2020 Jun 11;2020:6973636. doi: 10.1155/2020/6973636 (PMC7306075; doi:10.1155/2020/6973636)

**Supplementary Figure 1**

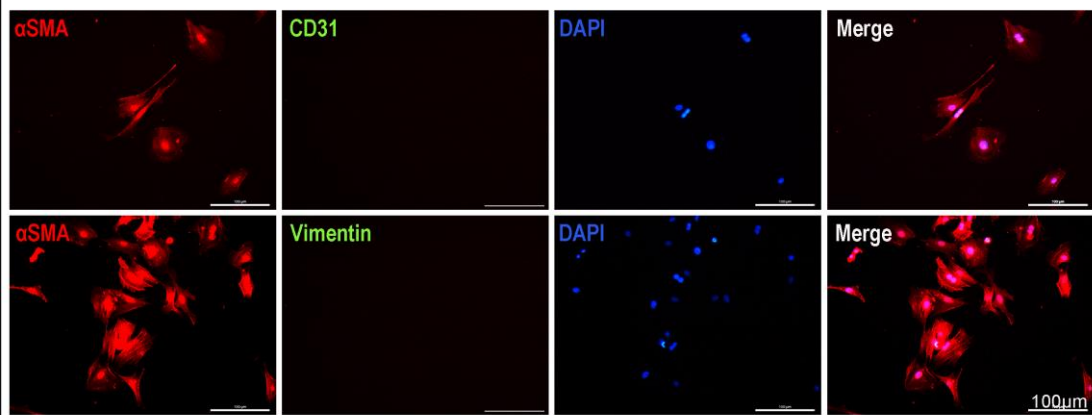

**Supplementary Figure 2**

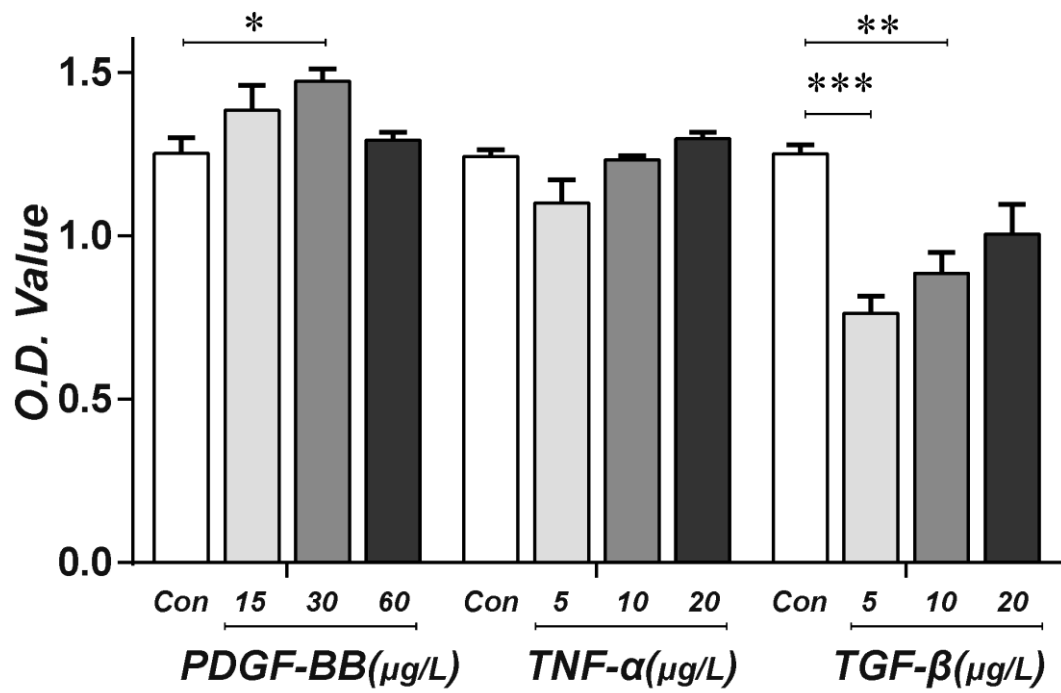

Supplement: Supplementary Materials — Supplementary Figure 1: PASMC isolation and identification. Supplementary Figure 2: PASMCs were starved for 24 h following indicated stimulation methods. [file 6973636.f1.pdf]
